# 

**Published:** 1879-07

**Authors:** 


### BOOKS AND PAMPHLETS RECEIVED.

Lecture on Electricity in its Relation to Medicine and Surgery. By A. D. Rockwell, A.M., M.D. Cl., pp. 99. 1879. New York: W. Wood & Co. Chicago: W. T. Keener.

The Twelfth Annual Report of the Health Department of the Honorable Common Council of the City of Cincinnati, for the year ending Dec. 31, 1879.

The Seventh Annual Report of the State Board of Health of Minnesota, Jan., 1879.

Posological Tables, including all the Officinal and Most Frequently Employed Unofficinal Preparations. By Chas. Rice. Cl., pp. 95. 1879. New York: Wm. Wood & Co. Chicago: W. T. Keener.

Ophthalmic Out-Patient Practice. By Chas. Higgins, F.R.C.S. Second edition. Cl., pp. 116. 1879. Philadelphia: Lindsay & Blakiston. Chicago: Jansen, McClurg & Co.

Diseases of the Throat and Nasal Passages — A Guide to the Diagnosis and Treatment of Affections of the Pharynx and Œsophagus, Trachea, Larynx and Nares. By J. S. Cohen. Second edition, revised and amended. Cl., pp. 42. 1879. New York: Wm. Wood & Co. Chicago: W. T. Keener.

The Laws of Therapeutics: or The Science and Art of Medicine. By Jos. Kidd, M.D Cl., pp. 196. Philadelphia: Lindsay & Blakiston. Chicago: Jansen, McClurg & Co.

Handbook of Diagnosis and Treatment of Diseases of the Throat and Nasal Cavities. By Car. Seiler. Cl., pp. 156. 1879. Philadelphia: H. C. Lea. Chicago: Jansen, McClurg & Co.

Hearing and How to Keep it. By Chas. H. Burnett, M.D. 1879. Cl., pp. 152. Philadelphia: Lindsay & Blakiston. Chicago: Jansen, McClurg & Co.

Fistula, Hæmorrhoids, Painful Ulcer, Stricture, Prolapsus and Other Diseases of the Rectum: Their Diagnosis and Treatment. By Wm. Arlingham. Third edition, partly rewritten. Cl., pp. 325, 1879. Philadelphia: Lindsay & Blakiston. Chicago: Jansen, McClurg & Co.

Transactions of the American Gynæcological Society. Vol. III, for the year 1878. Cl., pp. 472. Boston: Houghton, Osgood & Co. Chicago: Jansen, McClurg & Co.

Diseases of the Intestines. By John S. Bristowe, M.D., J. R. Wardell, M.D., J. W. Bigbie, M.D., S. O. Habershon, T. B. Curling, F.R.S., and W. H. Ransom, M.D. Cl., pp. 243. 1879. New York: Wm. Wood & Co.

Minutes of the Medical Society of the County of New York, 1806 to 1878. Edited by A. E. M. Purdy, M.D. May. Part II.

Seventh Annual Report of the Secretary of State of the State of Michigan, relating to the Register and Return of Births, Marriages, and Deaths for the year 1873. Lansing, Mich.

Atlas of Histology. By E. Klein, M.D., F.R.S., and E. Noble Smith, F.R.C.P., M.R.C.S. Parts I, II and III. Philadelphia: J. B. Lippincott & Co. Chicago: W. T. Keener.

Bibliotheca Dermatologica, Catalogue of Cutaneous Literature in the Library of H. G. Piffard, M.D.

Alternating Anterior and Posterior Version of the Uterus. By S. C. Bussy, M.D. Reprint from Vol. III, Gynæcological Transactions.

Address on State Medicine and Medical Organization. By Stanford A. Chaille, A.M., M.D.

Minutes of the Meeting of Organization and Proceedings of the Sanitary Council of the Mississippi Valley.

Further Contributions to the Treatment of Lupus. By Henry G. Piffard. Reprint from *Med. Record*, April 5, 1879.

On Spasmodic Stricture of the Urethra. A Reply to D. F. N. Otis. By H. B. Sands, M.D.

Transactions of the American Dental Association, commencing Aug. 6, 1878.

The Perihelia Crisis. By Richard Mansil, Rock Island, Ills.

Seventh Registration Report Concerning Vital Statistics of Michigan. General Remarks and Statements.

Other Symptoms of Nervous Exhaustion. By G. M. Beard, A.M., M.D. Reprint from the *Journal of Nervous and Mental Diseases*, April, 1879.

Conclusions from the Study of One Hundred and Twenty-five Cases of Writer's Cramp and Allied Affections. By G. M. Beard, M.D. Reprint from *Med. Record*, March 15, 1879.

---

DR. TILBURY FOX, of London, is dead. He was the author of a treatise on skin diseases which made his name known to many Americans, and whose merits certainly gained for its author a deserved eminence. Dr. Fox's death will be deeply felt by his large circle of warm friends.

### ANNOUNCEMENTS FOR THE MONTH.

### SOCIETY MEETINGS.

Chicago Medical Society—Mondays, July 7 and 21.

West Chicago Medical Society—Mondays, July 14 and 28.

### CLINICS.

MONDAY.

Eye and Ear Infirmary—2 p. m., Ophthalmological, by Prof. Holmes; 3 p. m., Otological, by Prof. Jones.

Mercy Hospital—1:30 p. m., Surgical, by Prof. Andrews.

Rush Medical College—2 p. m., Dermatological and Venereal, by Prof. Hyde; 3 p. m., Medical, by Dr. Bridge.

Woman's Medical College—2 p. m., Dermatological, by Dr. Maynard.

TUESDAY.

Cook County Hospital—2 to 4 p. m., Medical and Surgical Clinics.

Mercy Hospital—1:30 p. m., Medical, by Prof. Hollister.

WEDNESDAY.

Chicago Medical College—1:30 p. m., Eye and Ear, by Prof. Jones.

Rush Medical College—3:30 to 4:30 p. m., Diseases of the Chest, by Dr. E. Fletcher Ingals.

THURSDAY.

Chicago Medical College—1:30 p. m., Medical, by Prof. Quine.

Rush Medical College—3 p. m., Diseases of the Nervous System, by Prof. Lyman.

Eye and Ear Infirmary—2 p. m., Ophthalmological, by Dr. Hotz.

FRIDAY.

Cook County Hospital—2 to 4 p. m., Medical and Surgical Clinics.

Mercy Hospital—1:30 p. m., Medical, by Prof. Davis.

SATURDAY.

Rush Medical College—2 p. m., Surgical, by Prof. Gunn.

Chicago Medical College—2 p. m., Surgical, by Prof. Isham; 3 p. m., Neurological, by Prof. Jewell.

Woman's Medical College—11 a. m., Ophthalmological, by Dr. Montgomery.

Daily Clinics, from 2 to 4 p. m., at the Central Free Dispensary, and at the South Side Dispensary.

